# Metformin impairs Rho GTPase signaling to induce apoptosis in neuroblastoma cells and inhibits growth of tumors in the xenograft mouse model of neuroblastoma

**DOI:** 10.18632/oncotarget.2606

**Published:** 2014-10-21

**Authors:** Ambrish Kumar, Nadia Al-Sammarraie, Donald J. DiPette, Ugra S. Singh

**Affiliations:** ^1^ Department of Pathology, Microbiology and Immunology, University of South Carolina, Columbia, SC, USA; ^2^ Department of Internal Medicine, School of Medicine, University of South Carolina, Columbia, SC, USA

**Keywords:** Anticancer drug, Cell death, Metformin, Mitogen-activated protein kinase (MAPK), Neuroblastoma, Rho GTPases

## Abstract

Metformin has been shown to inhibit tumor growth in xenograft rodent models of adult cancers, and various human clinical trials are in progress. However, the precise molecular mechanisms of metformin action are largely unknown. In the present study we examined the anti-tumor activity of metformin against neuroblastoma, and determined the underlying signaling mechanisms. Using human neuroblastoma xenograft mice, we demonstrated that oral administration of metformin (100 and 250 mg/kg body weight) significantly inhibited the growth of tumors. The interference of metformin in spheroid formation further confirmed the anti-tumor activity of metformin. In tumors, the activation of Rac1 (GTP-Rac1) and Cdc42 (GTP-Cdc42) was increased while RhoA activation (GTP-RhoA) was decreased by metformin. It also induced phosphorylation of JNK and inhibited the phosphorylation of ERK1/2 without affecting p38 MAP Kinase. Infection of cells by adenoviruses expressing dominant negative Rac1 (Rac1-N17), Cdc42 (Cdc42-N17) or constitutively active RhoA (RhoA-V14), or incubation of cells with pharmacological inhibitors of Rac1 (NSC23766) or Cdc42 (ML141) significantly protected neuroblastoma cells from metformin-induced apoptosis. Additionally, inhibition of JNK activity along with Rac1 or Cdc42 attenuated cytotoxic effects of metformin. These studies demonstrated that metformin impairs Rho GTPases signaling to induce apoptosis via JNK pathway.

## INTRODUCTION

Neuroblastoma is solid tumor of the postganglionic sympathetic nervous system, arises in the adrenal glands and spreads to various organs such as liver, bone, lymph nodes, neck and chest [1]. It is the most common cancer in babies younger than one and second most common tumors in children. In the United States of America, approximately 700 children are diagnosed with neuroblastoma each year. It accounts for 7% of all childhood cancers (Cancer Facts & Figures 2014, Atlanta, GA: American Cancer Society), and is responsible for 15% of all cancer deaths in children younger than 15 years. The five-year survival rate for children with high-risk neuroblastoma is only about 30% - 50% [2]. Neuroblastoma tumor consists of heterogeneous populations of cells differing at morphological and biochemical levels. The alterations in karyotype and cytogenetic characteristics, such as genomic amplification of MYCN gene, mutations in tumor suppressor genes and rearrangement or deletion in chromosomes, develop drug resistance and help neuroblastoma tumors to escape most available therapies [3-5]. Thus, the mortality rate remains high for these patients. Therefore, search for novel therapeutic compounds that can work on a wide range of neuroblastoma cells are desperately needed for neuroblastoma therapy.

Metformin (*N*’,*N*’-dimethylbiguanide) is widely used as a first line therapy for the treatment of type 2 diabetes [6, 7]. In recent years, oncologists are paying considerable attention to metformin as some population-based studies showed a low cancer incidence and mortality among diabetic patients treated with metformin [8-13]. These studies give insight for scientists to study the antineoplastic activity of metformin [14, 15]. Metformin efficiently inhibits tumor growth in xenograft models of various adult cancers [16-21] and human clinical trials of metformin are currently in progress (www.clinicaltrials.gov), however, its anti-tumor activity against childhood cancers is not well known.

The aim of the present study was to test the antitumor activity of metformin against neuroblastoma and delineate the underlying signaling mechanisms. Using SH-SY5Y and SK-N-BE(2) xenograft neuroblastoma mice models, our *in vivo* results for the first time demonstrated that metformin significantly inhibits the growth of tumors. Metformin alters activation of Rho-GTPases (RhoA, Rac1 and Cdc42), and affects MAP kinases phosphorylation, which in turn induces apoptosis. By expressing constitutively active or dominant negative forms of Rho GTPases, and by using specific inhibitors of Rac1, Cdc42, and JNK, we further confirmed the role of impairments in Rho GTPase signaling in mediating metformin effects on the survival of neuroblastoma cells.

## RESULTS

### Metformin inhibits neuroblastoma growth *in vivo*

**FIGURE 1:**
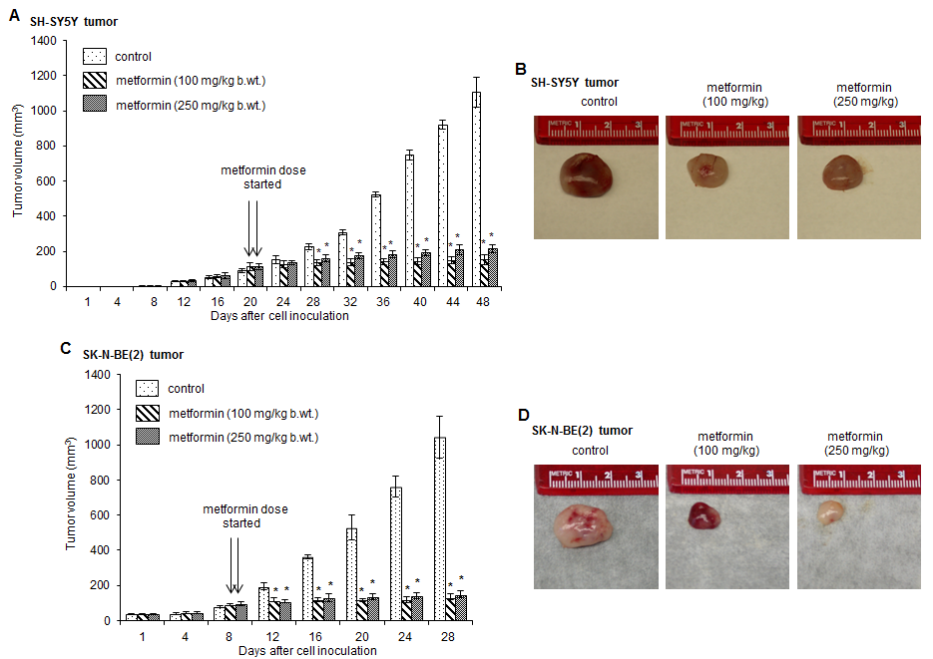
Metformin inhibits the growth of tumors in xenograft model Athymic mice were injected with SH-SY5Y cells (A and B) or SK-N-BE(2) cells (C and D) subcutaneously in the flank region. When the palpable tumor size was ~100 mm^3^, mice were fed daily with metformin (100 mg/kg b.wt. and 250 mg/kg b.wt.) by oral gavage (on day 8 in SK-N-B(2) xenograft mice and on day 20 in SH-SY5Y xenograft mice after cell inoculation, as indicated by arrows). Tumor volume was measured after every 4 days and plotted. At the end of experiments, tumors were collected and photographed. *p < 0.05 *vs* control.

To assess the anti-tumor activity of metformin against neuroblastoma, we tested two neuroblastoma xenograft mice models. The localized subcutaneous neuroblastoma tumors were generated by injecting either SH-SY5Y cells or SK-N-BE(2) cells. Metformin treatment (100 or 250 mg/kg/mice/day by oral gavage) was started when the palpable tumor volume was reached ~100 mm^3^ (on day 8 in SK-N-BE(2) xenograft mice and on day 20 in SH-SY5Y xenograft mice after cell inoculation). The size of tumors was measured on every fourth day and plotted. Figure 1A and indicated that the average tumor size in animals receiving metformin (100 or 250 mg/kg b.wt.) was significantly smaller compared to tumors in metformin-untreated mice (*p < 0.05 *vs* control). After 28 days of metformin treatments, the average tumor volume was ~155 ± 28.86 mm^3^ (at 100 mg/kg metformin dose), ~215 ± 23.8 mm^3^ (at 250 mg/kg metformin dose) and ~1105 ± 83.73 mm^3^ in metformin-untreated tumors from SH-SY5Y xenograft mice. Similarly, in SK-N-BE(2) neuroblastoma xenograft mice, the average size of tumors in control, metformin 100 mg/kg and metformin 250 mg/kg was 1043 ± 117.07 mm^3^, 132 + 17 mm^3^, 149 ± 20.02 mm^3^, respectively (*p < 0.05 *vs* control; Fig. 1C and D). Metformin at lower doses (50 mg/kg b.wt.) did not affect tumor growth (data not shown). At the end of experiments we did not observe the toxicity of metformin as all metformin-fed mice were survived with no complications in physical appearance.

### Metformin promotes apoptosis in tumors

**FIGURE 2:**
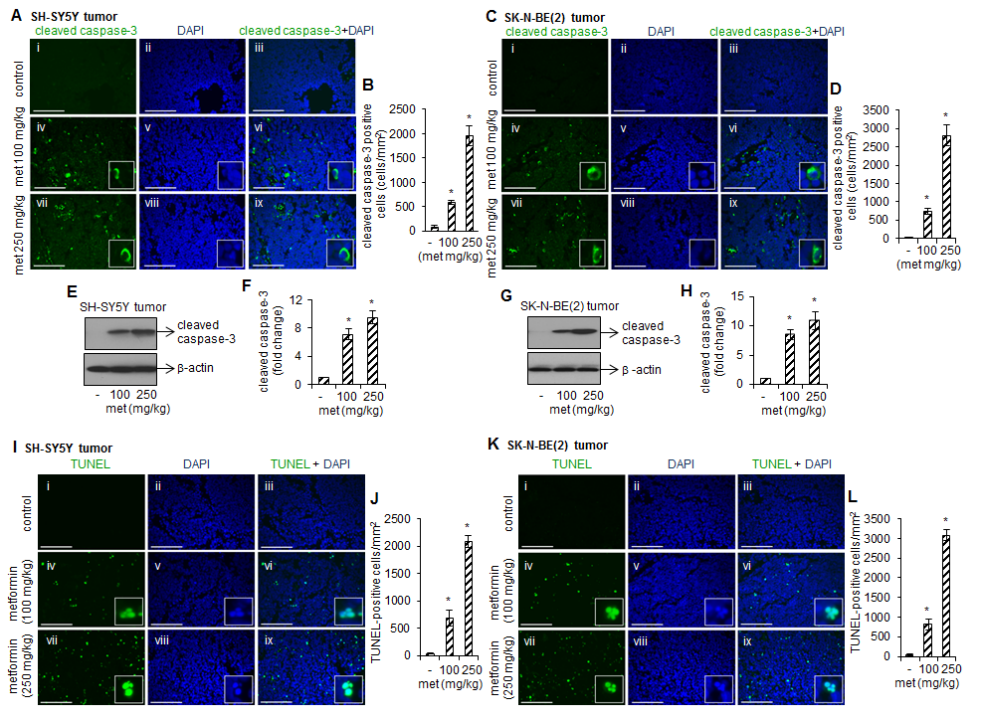
Metformin induces activation of caspase-3 and DNA fragmentation in tumors Representative immunofluorescence images showing cleaved caspase-3 staining (green) in SH-SY5Y tumors (A) and SK-N-BE(2) tumors (C). Nuclei were counterstained with DAPI (blue). The cytoplasmic cleaved caspase-3 staining (merged enlarged images) was observed in tumors treated with metformin. Scale= 100 μm. Cleaved caspase-3 positive cells were quantitated from 15 random fields/tissue sections by ImageJ software and average from ten tumor tissues from control and experimental mice was plotted (B and D). Values represent mean ± SD. *p < 0.05 *vs* control. Western blots showing cleaved caspase-3 protein level in metformin-treated and –untreated SH-SY5Y (E and F) and SK-N-BE(2) tumors (G and H). Blots were reprobed with β-actin for loading difference. Band intensity was measured by ImageJ and the ratio of cleaved caspase-3/β-actin was plotted (F and H). The values represent mean ± SD from ten animals from control and experimental groups. *p < 0.05 *vs* control. (I-L) Fluorescence images showing TUNEL staining (green) in SH-SY5Y tumors and SK-N-BE(2) tumors. Nuclei were counterstained with DAPI (blue). The TUNEL-staining (an indicator of DNA fragmentation) was detected exclusively in nucleus (merged enlarged images). Scale= 100 μm. The TUNEL-positive cells were counted in 15 random fields/tissue sections by ImageJ software and average from ten tumor tissues from control and experimental mice was plotted (J and L). Values represent mean ± SD. *p < 0.05 *vs* control.

To determine if metformin-inhibited tumor growth were resulted from apoptotic cell death, we performed immunohistochemistry by staining paraffin-embedded tumor sections with antibody specific for active cleaved form of caspase-3. Cleaved caspase-3 positive cells (an indicator of apoptosis) were quantitated by ImageJ software and plotted. The representative immuno-fluorescence images demonstrated the significant increased numbers of cleaved caspse-3 positive cells (green) in tumors from metformin-fed (100 or 250 mg/kg) SH-SY5Y (Fig. 2A iv and vii, and B) and SK-N-BE(2) xenograft mice (Fig. 2C iv and vii, and D) compare to metformin-untreated tumors (Fig. 2A i and C i; *p < 0.05 *vs* control). The cleaved caspse-3 signals were exclusively detected in cytoplasm (enlarged images) and did not overlapped with nucleus (Fig. 2A vi and ix; and Fig. 2C vi and ix). Western blots using total cell proteins extracted from these tumors showed that metformin at 100 mg/kg dose and 250 mg/kg dose increased cleaved caspase-3 level by ~7 fold and ~9 fold, respectively, compare to control SH-SY5Y tumors (*p < 0.05 *vs* control, Fig. 2E and F). Similar metformin-induced activation of caspase-3 was observed in SK-N-BE(2) tumors (Fig. 2G and H). Thus the presence of cleaved caspse-3 signals indicates that metformin promotes apoptotic cell death to reduce tumor size.

We next examined metformin-induced DNA fragmentation by TUNEL assay. The TUNEL positive cells (an indication of DNA fragmentation) were counted by ImageJ software and plotted. The representative images in Fig. 2I-L showed that compare to control, metformin at 100 mg/kg and 250 mg/kg increased the number of TUNEL positive cells (green) in SH-SY5Y (Fig. 2I and J) and SK-N-BE(2) tumors (Fig. 2K and L) (*p < 0.05 *vs* control). The staining for nuclei (blue) and fragmented DNA (TUNEL-positive staining) overlapped in a single cell (merged images; Fig. 2I; and Fig. 2K) suggest that metformin promotes apoptosis in these cells.

### Metformin inhibits spheroid formation and reduces cell viability *in vitro*

**FIGURE 3:**
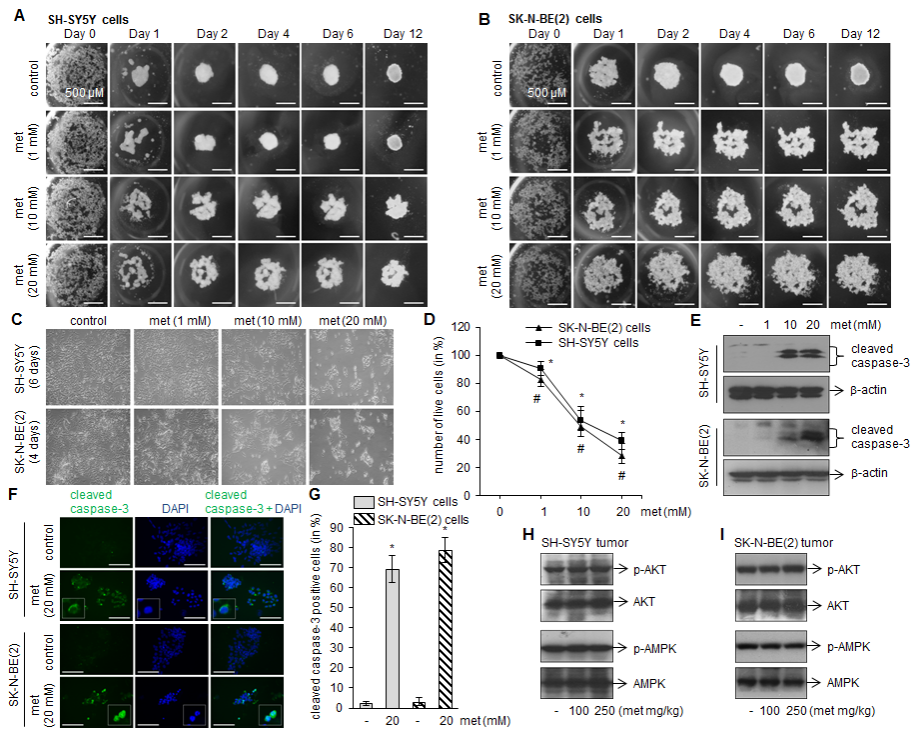
Metformin inhibits the neuroblastoma spheroid formation and induces apoptotic cell death *in vitro* (A and B) Representative images showing progression of spheroid formation in presence or absence of metformin in *in vitro* hanging drop assay. Scale= 500 μm. Experiments were performed four times independently. (C) Phase contrast images showing cell morphology after metformin treatments (1, 10 and 20 mM). After 4 days (for SK-N-BE(2)) and 6 days (for SH-SY5Y) of metformin treatments cells were photographed (C), live cells were counted by trypan blue and plotted (D). The values represent mean ± SD from eight independent experiments. For SH-SY5Y, *p < 0.05 *vs* control; for SK-N-BE(2), #*p* < 0.05 *vs* control. Western blots (E) and immunofluorescence (F) for cleaved caspase-3 was performed after 4 days (SK-N-BE(2) cells) and 6 days (SH-SY5Y) metformin exposure. DAPI was used to stain nuclei (blue). Scale= 100 μm. Cleaved caspase-3 positive cells (green) were counted in ten random fields by imageJ and plotted (G). The experiments were performed three times independently. *p < 0.05 *vs* control. Representative Westen blots showing levels of phosphorylated and total form of AMPK and AKT in SH-SY5Y tumor (H) and SK-N-BE(2) tumor (I). The blots are representative of ten tumor samples from control and experimental mice.

Since metformin inhibits tumor growth in xenograft mice (Fig. 1), we further tested if metformin also interfere in the initiation of tumor formation by performing *in vitro* hanging drop assay[22, 23]. The formed spheroids represent an *in vitro* 3-D tissue structure that mimics *in vivo* tumor tissue organization and microenvironment and better reflect cancer cells in their native, *in vivo,* environment. Under control conditions, both SH-SY5Y and SK-N-BE(2) cells grown in hanging drop culture start aggregating at the bottom of the droplet. On the following days, the shape of the aggregates became rounder and smoother with a gradual decrease in the radius of the spheroid, and form compact cell clusters resulted from higher cell–cell cohesion. However, metformin dose-dependently disrupt the large single-cluster formation as a result cells become loosely attached to each other and unable to form compact spheroids (Fig. 3A and B).

To examine metformin effects on neuroblasotma cell viability, SH-SY5Y and SK-N-BE(2) cells were grown in presence of metformin (1, 10 and 20 mM), and on subsequent days cells were photographed and counted by trypan blue cell viability assay. The phase contrast images taken on day 4 (SK-N-BE(2) cells) and day 6 (SH-SY5Y cells) demonstrated that metformin promotes cells death (as evaluated by rounded-clumped cell morphology; Fig. 3C), and the IC50 was found in range of 10–12 mM for both cell lines tested (Fig. 3D). Metformin at and above 10 mM concentration significantly activated caspase-3 as observed in Western blots (Fig. 3E) and immunofluorescence microscopy for cleaved caspase-3 (Fig. 3F and G; *p < 0.05 *vs* control).

In order to delineate metformin-induced apoptotic mechanism in neuroblastoma, we first examined the activation of AKT and AMPK which are well known intracellular mediators stimulated by metformin. Our western blots demonstrated that metformin at either dose (100 or 250 mg/kg) did not phosphorylate AKT and AMPK in tumor samples collected from xenograft mice (Fig. 3H and I). Similarly no phosphorylation of these proteins was observed in *in vitro* culture treated with metformin (1, 10 and 20 mM; data not shown). These results indicated that the cytotoxic effects of metformin against neuroblastona are AKT- and AMPK-independent, and is supported by others in different cancers [24, 25].

### Metformin affects activation of MAP kinases

**FIGURE 4:**
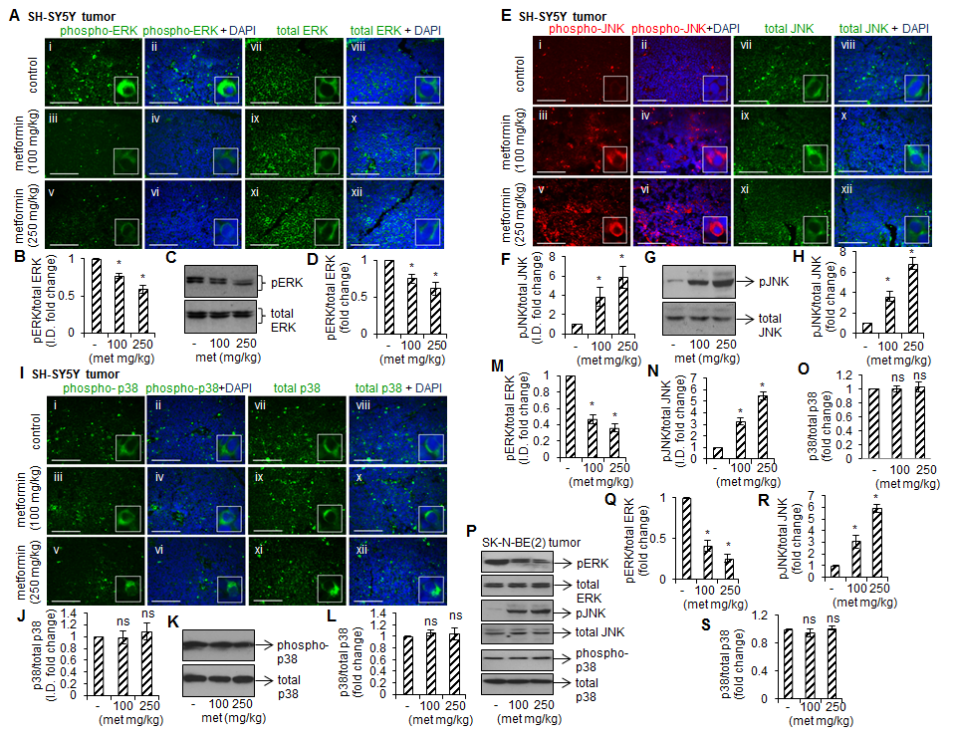
Metformin affects phosphorylation of MAP kinases Immunofluorescence images showing the staining of phospho-ERK and total ERK (A), phospho-JNK and total JNK (E), and phospho-p38 and total p38 (I) in SH-SY5Y tumors. Nuclei were counterstained with DAPI (blue). Scale= 100 μm. The fluorescence intensity from twelve random fields of each tissue section was measured using ImageJ and the ratio of phospho- and total levels (B, F, J) were plotted. The values represent mean ± SD from ten tumors from control and experiment groups. Representative Western blots showing phosphorylated and total form of ERK (C), JNK (G) and p38 (K) in SH-SY5Y tumors. The protein band intensity was measured by ImageJ, and the ratio of phosphorylated and total form was plotted (D, H, L). The values are mean ± SD from ten tumors from control and experiment mice. Similar to SH-SY5Y tumors, the immuno-histochemistry for ERK, JNK and p38 was carried out in SK-N-BE(2) tumors. The fluorescence intensity was measured in eight random field/tissue section by ImageJ, and the ratio phosphorylated and total levels were plotted (M-O). The values are mean ± SD from ten tumors from control and experiment mice. (P) Representative Western blots of phosphorylated and total ERK, JNK and p38 in SK-N-BE(2) tumors. The protein band intensity was measured by ImageJ, and the ratio of phosphorylated and total was plotted (Q-S). Results are mean ± SD from ten tumors from control and experiment groups. *p < 0.05 *vs* control. ns= not significant to control.

Mitogen-activated protein kinases (MAPKs; ERK1/2, JNK and p38) are serine/threonine specific kinases and their role in cell survival, proliferation and apoptosis is well known [26]. To test if inhibitory effects of metformin are mediated by MAP kinases, we measured activation (phosphorylation) of ERK1/2, JNK and p38 by immunohistochemistry and Western blotting. The representative immunofluoresence images showed the reduced levels of phospho-ERK1/2 in SH-SY5Y tumors from metformin-fed mice (100 and 250 mg/kg) (Fig. 4A i-vi). The fluorescence intensity for total ERK1/2 remained unchanged in metformin-treated and -untreated tumors (Figs. 4A-vii, ix and xi). The fluorescence intensities as measured by ImageJ programme indicated that compare to control the phospho-ERK/total-ERK ratio was ~30% and ~40% less at 100 mg/kg and 250 mg/kg doses in metformin-treated tumors, respectively (*p < 0.05 *vs* control, Fig. 4B). The observed reduced phosphorylation of ERK1/2 in tumors from metformin-fed mice in Western blots further confirmed our immunohistochemistry data (Fig. 4C and D).

In SH-SY5Y tumors, metformin (100 mg/kg and 250 mg/kg) increased JNK phosphorylation compare to control as detected by immunohistochemistry without changing total JNK level in any group (Fig. 4E). The ratio of phospho-JNK/total-JNK fluorescence intensity showed that metformin at these doses increased JNK phosphorylation by ~3.8 fold and ~5.8 fold, respectively, compare to control (*p < 0.05 *vs* control, Fig. 4F). The Western blots for JNK further confirmed our immunofluorescence observations (Fig. 4G and H). In contrast to ERK and JNK, we did not observe metformin-induced p38 phosphorylation in these tumors (Fig. 4I-L). Similar effect of metformin on phosphorylation of these MAPKs was observed in SK-N-BE(2) tumors (immunofluorescence data, Fig. 4M-O; and Western blot data, Fig. 4P-S).

### Metformin impairs the activation of Rho GTPases

**FIGURE 5:**
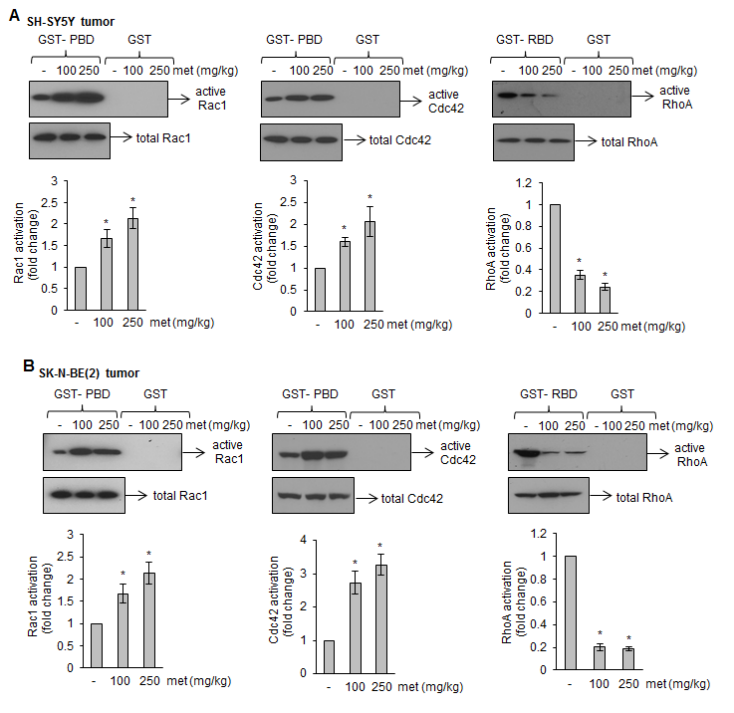
Metformin affects Rac1, Cdc42, and RhoA activation in tumors (A and B) Western blots of affinity-precipitated (GST-PBD and GST-RBD) tumor lysates from metformin-treated or untreated xenograft mice. GST-PBD was used to precipitate active form of Rac1 and Cdc42 while GST-RBD was used to precipitate active form of RhoA. As a control, pull-down assay by GST alone was carried out. Corresponding bar diagrams showing the activation of these GTPases as calculated by densitometric scanning of signals described in Methods section. Results are average from eight tumors from control and experiment mice. *p < 0.05 *vs* control.

**FIGURE 6:**
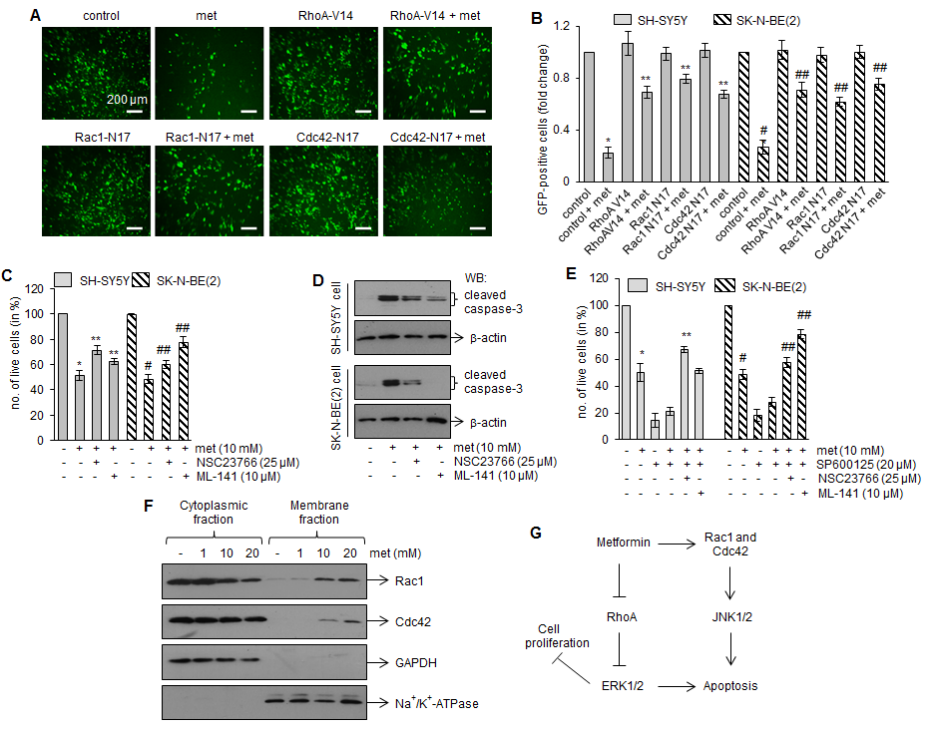
Rho GTPase inhibitors attenuate metformin effects on the survival of neuroblastoma cells (A) Fluorescence images showing the signals of GFP-Rac1, GFP-Cdc42 and GFP-RhoA in SH-SY5Y cells. Cells were infected with adenoviruses expressing GFP-fused dominant–negative Rac1-N17 and Cdc42-N17, and constitutively active RhoA-V14. GFP alone were used as control. After 24 h infection, cells were treated with metformin (10 mM) and further grown for 6 days and photographed. Scale= 200 μm. Number of GFP-containing cells (green) was counted from 10 random fields and plotted (B). Similar adenovirus experiments were performed with SK-N-BE(2) cells, and number of GFP-positive cells was counted after 4 days of metformin (10 mM) treatment and plotted (B). Results are mean ± SD from four independent experiments. *p < 0.05 *vs* control, **p < 0.05 *vs* metformin 10 mM, #*p* < 0.05 *vs* control, ##*p* < 0.05 *vs* metformin 10 mM. (C) Bar diagram showing number of live cells after treatments with metformin (10 mM) alone or in combination with Rac1 inhibitor (NSC23766; 25 μM) or Cdc42 inhibitor (ML141; 10 μM). After 4 days (SK-N-BE(2)) or 6 days (SH-SY5Y) treatments, number of live cells were counted by trypan blue. The experiments were performed four times and values represent mean ± SD. *p < 0.05 *vs* control, **p < 0.05 *vs* metformin 10 mM, #*p* < 0.05 *vs* control, ##*p* < 0.05 *vs* metformin 10 mM. Total cell proteins were extracted from these treatment groups and Western blotting for cleaved caspase-3 was performed (D). Blots are representative of four independent experiments. (E) Bar diagram showing number of live cells after treatments with JNK inhibitor (SP600125; 20 μM) alone or in combination with Rac1 inhibitor (NSC23766; 25 μM), Cdc42 inhibitor (ML141; 10 μM) and metformin (10 mM) for 4 days (SK-N-BE(2) or 6 days (SH-SY5Y cells). Each bar represents mean ± SD of six independent experiments. *p < 0.05 *vs* control, **p < 0.05 *vs* metformin 10 mM, #*p* < 0.05 *vs* control, ##*p* < 0.05 *vs* metformin 10 mM. (F) Western blots showing Rac1 and Cdc42 levels in cytoplasmic and membrane fractions prepared from SH-SY5Y cells after 6 days metformin treatments. Antibodies against glyceraldehyde 3-phosphate dehydrogenase (GAPDH) and Na^+^/K^+^-ATPase were used as cytoplasmic and plasma membrane protein markers, respectively. Results are representative of three independent experiments. (G) Putative model for metformin signaling in neuroblastoma cells. Metformin activates Rac1 and Cdc42, and inhibits RhoA in neuroblastoma cells. The activated Rac1 and Cdc42, in turn, promote phosphorylation of JNK, while inhibition of RhoA reduces ERK phosphorylation. These effects initiate apoptotic cell death in neuroblastoma cells and inhibit tumor growth in xenograft mice model of neuroblastoma.

In response to various stimuli, Rho GTPases (such as RhoA, Rac1 and Cdc42) regulate MAP kinase activation that, in turn, induces apoptosis [27, 28]. Hence, we tested whether Rho GTPases activation were involved in metformin-induced apoptotic signaling. Our GST-pull down assays revealed that metformin increased Rac1 and Cdc42 activation, as indicated by strong GTP-Rac1 and GTP-Cdc42 signals, in both SH-SY5Y and SK-N-BE(2) tumors (Fig. 5A and B). In these tumors, metformin treatments inhibited RhoA activation, as indicated by weak GTP-RhoA signals compare to control samples (Fig. 5A and B). To further confirm that altered activated Rho GTPases played a critical role in metformin-induced cell death, we infected neuroblastoma cells with adenoviruses expressing either GFP-fused constitutively active or dominant negative forms of RhoA, Rac1 and Cdc42, and treated with metformin (10 mM) for 4 days (SK-N-BE(2)) or 6 days (SH-SY5Y). Cells were photographed; GFP-positive cells were counted and plotted. Overexpression of the constitutively active RhoA (RhoA-V14), dominant negative Rac1 (Rac1-N17) and Cdc42 (Cdc42-N17) significantly increased the viability of metformin-treated cells (Fig. 6A and B). In another experiment we incubated neuroblastoma cells with pharmacological inhibitors specific for Rac1 (NSC23766; 25 μM) or Cdc42 (ML141; 10 μM) one hour prior to metformin (10 mM) treatment, and cell viability was accessed after 4 days (SKN-BE(2) cells) and 6 days (SH-SY5Y cells) of treatments by trypan blue. Bar diagram in figure 6C showed that exposure of Rac1 inhibitor (NSC23766) and Cdc42 inhibitor (ML141) significantly reduced the cytotoxicity of metformin in both cell lines tested. Western blot analyses carried out with these cells (Fig. 6D) demonstrated that addition of NSC23766 (25 μM) or ML-141(10 μM) to metformin (10 mM) treated SH-SY5Y and SK-N-BE(2) cells reduced the activation of cleaved caspase-3 (lane 3 and 4) compared to metformin (10 mM) alone (lane 2). These results further indicated that inhibition in Rac1 and Cdc42 rescue SH-SY5Y and SK-N-BE(2) cells against metformin–induced apoptosis.

Since signaling by Rac1 and Cdc42 is known to stimulate JNK pathway [29], we next examined whether metformin induced JNK phosphorylation is resulted from Rac1/Cdc42 activation in our case. Neuroblastoma cells were incubated with JNK inhibitor (SP600125; 20 μM) with metformin (10 mM) in presence of Rac1 inhibitor (NSC23766; 25 μM) or Cdc42 inhibitor (ML141; 10 μM), and cell viability was measured. Exposure of JNK inhibitor (SP600125; 20 μM) alone or in combination with metformin (10 mM) markedly inhibited the growth of SH-SY5Y and SK-N-BE(2) cells (Fig. 6E). However, addition of Rac1 inhibitor (NSC23766; 25 μM) or Cdc42 inhibitor (ML141; 10 μM) in SP600125 + metformin treated cells significantly increased the cell viability in these cells. These data suggested that JNK plays secondary role in metformin-induced cell death and it is downstream to Rac1 and Cdc42 in signaling pathways.

Upon activation, Rho GTPases interact with their effectors (PAK1 for Rac1 and Cdc42, and ROCK-2 for RhoA), translocate to plasma membranes and regulate MAP Kinase-mediated apoptosis [30-32]. Since metformin activates Rac1 and Cdc42, we determined the translocation of these GTPases to the plasma membranes. Western blots in Fig. 6F demonstrated that exposure of metformin promoted translocation of Rac1 (upper panel) and Cdc42 (lower panel) to the plasma membranes in SH-SHY5Y cells. These results indicated that Rho GTPase signaling is involved in inhibitory effects of metformin on neuroblastoma.

## DISCUSSION

The present study reports the antitumor effects of metformin against neuroblastoma. Using two human neuroblastoma cell lines of different genotype- SH-SY5Y (MYCN-non amplified) and SK-N-BE(2) cells (MYCN-amplified, multi-drug resistant), our *in vivo* studies demonstrate that metformin significantly inhibits the growth of tumors in xenograft mice (Fig. 1). Metformin reduces viability of neuroblastoma cells (Fig. 3C-D) and its interference with the spheroid formation in 3-D cultures (Fig. 3A-B) further confirms the anti-neoplastic effect of metformin against neuroblastoma. These data also indicate that SK-N-BE(2) cells (MYCN-amplified and harbor p53 mutation) are more sensitive to metformin compared to SH-SY5Y cells. It is known that MYCN-amplification and p53-mutation is associated with neuroblastoma progression and drug resistance [33-35]. MYCN, a member of the myc family of proto-oncoproteins, regulates the expression of genes involved in the cell cycle, DNA damage and apoptosis, and overexpression of MYCN results in neuroblastoma development [36]. However, MYCN downregulation induces apoptosis in neuroblastoma cells [37]. p53 is a tumor suppressor protein and the presence of nonfunctional mutated p53 fails to induce apoptosis after DNA damage and makes cells resistant to cytotoxic agents. Our observation that metformin is more selective to p53-mutated cells is also supported by another report showing metformin selectively inhibits tumor growth of p53-deficient colon cancer cells *in vivo* [19], and signifies that metformin exhibits cytotoxic effects irrespective of genetic background in neuroblastoma cells.

The inhibitory effects of metformin on neuroblastoma growth resulted from the caspase-mediated cell death (Fig. 2A-H). The presence of cleaved caspase-3 signals and apoptotic nuclei in tumors from metformin-treated mice (Fig. 2) confirm that caspase activation by metformin triggers apoptotic cell death pathways in neuroblastoma cells. These results are in agreement with other reports showing metformin-induced cell death occurs via activation of caspase cascade [24, 38, 39].

Rho GTPases (RhoA, Rac1 and Cdc42), a family of small G-proteins, act as molecular switches to regulate a variety of cellular functions including cell division, motility, cell adhesion and cell survival [40]. These GTPases either exist as a GTP-bound active form or an inactive GDP-bound form. The aberrant expression and/or activity of Rho GTPases is associated with the progression of various tumors [41]. Interestingly, using a GST-pull down assay, we show that metformin significantly increases the levels of the active form of Rac1 (GTP-Rac1) and Cdc42 (GTP-Cdc42) while decreasing RhoA activation (GTP-RhoA) in tumors (Fig. 5). Incubation of pharmacological inhibitors of Rac1 (NSC23766) and Cdc42 (ML141), expression of dominant–negative Rac1-N17 and Cdc42-N17, or constitutively active RhoA-V14 significantly protected cells from metformin-induced apoptosis (Fig. 6A-D). These gain-in and loss-of-function experiments clearly suggest the importance of these Rho-family proteins in the apoptotic effects of metformin.

We further observed that metformin affects the phosphorylation of MAP kinases (ERK, JNK and p38) in SK-N-BE(2) and SH-SY5Y tumors. The phosphorylation of ERK was decreased, JNK was increased and p38 remained unchanged in tumors from metformin-fed mice (Fig. 4). Metformin-induced apoptosis through ERK and JNK is reported in lung and breast cancers [24, 42]. Blocking JNK activity by its pharmacological inhibitor (SP600125) alone was not able to attenuate the inhibitory effect of metformin on cell viability; however, addition of Rac1 inhibitor (NSC23766) or Cdc42 inhibitor (ML141) along with SP600126 (JNK inhibitor) significantly protected neuroblastoma cells against metformin toxicity (Fig. 6E). These results suggest that JNK is downstream to Rac1 and Cdc42 in signaling pathways. This notion is supported by the previous observations that Rho GTPases regulate gene expression through the activation of kinase cascades leading to enhanced activity of MAPKs e.g., RhoA inhibition reduces cell proliferation and migration via ERK pathway while Rac1 and Cdc42 activation promotes apoptosis via JNK pathways [27-29]. Thus the ability of metformin to stimulate JNK suggests that these Rho GTPases promote the initiation of the apoptotic pathway via JNK, thus resulting in neuroblastoma cell death (Fig. 6G).

Mitochondria are the primary site of action of metformin, where it activates phosphorylation of AMPK that, in turn, inhibits mTOR pathways and induces cell death [43]. In addition, AMPK-independent actions of metformin have also been reported which include inhibition of mTOR signaling either through RAG GTPases [44], through enhancement of PRAS40-RAPTOR association in glioma [25], or through activation of JNK/p38 MAPK-mediated apoptotic pathway to inhibit lung cancer [24]. Our results show that metformin, independent of AMPK and AKT, inhibits neuroblastoma tumorigenesis through differential regulation of Rho GTPases. How metformin activates these GTPases is unclear. It might be possible that metformin affects posttranslational modifications of these Rho GTPases [45], or regulates Rho GTPase interacting proteins [46] to exhibit its apoptotic effects in neuroblastoma. In our studies, activation and translocation of Rac1 and Cdc42 to the plasma membranes by metformin (Fig. 5 and 6F) suggest the involvement of Rho GTPase interacting proteins in inhibitory effects of metformin.

In summary, our *in vivo* results report the inhibitory effect of metformin against neuroblastoma, and for the first time demonstrates the role of Rho GTPases in metformin-mediated apoptotic cell death. The cytotoxic effects of metformin against MYCN-nonamplified as well as MYCN-amplified multidrug resistant neuroblastoma cells further signify that metformin can be a promising drug candidate for neuroblastoma therapy. The anti-proliferative and apoptotic effects of metformin, even at millimolar concentrations, were not observed in undifferentiated human normal stem cells (e.g. umbilical cord-derived mesenchymal stem cells) [47], primary cortical neurons [48, 49], and in various normal endothelial cells such as human microvascular endothelial cells (HMEC-1) and primary endothelial cells from either human umbilical vein or bovine aorta [50]. These studies suggest the highly selective and cytotoxic effects of metformin on neuroblastoma cells. As metformin is cost effective and safe drug approved by United States Food and Drug Administration (FDA) to treat type 2 diabetes in children [51], the present study suggests that metformin can also be used as a novel therapeutic agent against pediatric cancers, especially neuroblastoma.

## METHODS

### Cell culture and treatments

Human neuroblastoma SK-N-BE(2) and SH-SY5Y cells (ATCC, Manassas, VA) were maintained in complete culture medium (Dulbecco's Modified Eagle's Medium, DMEM + 10% fetal bovine serum, FBS; Atlanta Biologicals, Lawrenceville, GA) at 37°C in a humidified incubator with 5% CO_2_. Stock solution of metformin (MP Biomedicals, Solon, OH) was freshly prepared in sterile triple distilled water before each experiment. Cells treated with equal volume of vehicle were used as control.

### *In vivo* mouse xenograft model

Six-week-old female homozygous nude mice (nu/nu; Crl:NU-Foxn1nu;Charles River Laboratories, Wilmington, MA) were housed per the recommendations and approvals of the Institutional Animal Care and Use Committee (IACUC) of the University of South Carolina. The animals were maintained at 25°C with a 12-h light/12-h dark cycle in laminar flow cabinets under specific pathogen–free conditions and given sterile water and food *ad libitum*.

We used neuroblastoma xenograft mice model using SH-SY5Y cells or SK-N-BE(2) cells. To develop localized subcutaneous tumors, 2 × 10^7^ cells were mixed 1:1 with matrigel (BD biosciences, San Jose, CA) to make 0.1 ml total volume and were injected subcutaneously into the right flank of mice (n= 72). The animal weight and general appearance were recorded regularly throughout the experiments. On every fourth day, tumor size was measured with calipers and tumor volume was calculated using the ellipsoid formula (length x width x height x 0.5). When tumor volume reached a palpable size of ~100 mm^3^, mice were divided into three groups- (i) control without metformin, n=12; (ii) with metformin at dose 100 mg/kg body weight/mice, n=12; and (iii) with metformin at dose 250 mg/kg body weight/mice, n=12. Metformin dissolved in sterile water (200 μl) was given daily by oral gavage. When the tumor reached terminal size (≥1000 mm^3^) in metformin-untreated group, mice from all groups were euthanized, tumors were harvested and weighed. Portions of tumor were fixed in 4% paraformaldehyde or snap frozen in liquid nitrogen for further use in biochemical assays.

### Protein extraction and Western blot analyses

For total protein isolation, tumor tissues or cells were lysed with cell lysis buffer (Cell Signaling Technology, Danvers, MA). Plasma membrane and cytoplasmic protein enriched fractions were prepared using plasma membrane protein extraction kit (BioVision Inc, Milpitas, CA). Equal amount of proteins were separated in SDS-polyacrylamide gel and analyzed by Western blotting [52]. Primary antibodies used are: cleaved caspase-3, phospho-ERK, phospho-JNK, phospho-p38, β-actin, and phospho- and total-forms of AKT and AMPK (Cell Signaling Technology), and total-ERK, total- JNK and total-p38 (Santa Cruz Biotechnology, Dallas, TX).

### Immunohistochemistry

Paraformaldehyde-fixed paraffin-embedded tumor sections (5 μm thick) were deparaffinized in xylene, rehydrated with sequential immersion in graded ethanol (100%, 95%, 85%, 70% and 50%). Antigen unmasking was carried out by boiling slides in 10 mM sodium citrate buffer, pH 6.0 at 95 °C for 30 min. After cooling down at room temperature, sections were permeabilized with 0.2% Triton X-100/PBS for 10 min, and were blocked with 10% immunoglobulin free-bovine serum albumin (IgG-free BSA; Jackson ImmunoResearch Laboratories, West Grove, PA) in 1x PBS for overnight at 4 °C. Sections were incubated with primary antibodies diluted in 5% IgG-free BSA/PBS (1:100 dilution) for overnight at 4 °C. Primary antibodies were detected with secondary antibodies conjugated with fluorescein isothiocyanate (FITC) or rhodamine (1:4000 dilution in 5% IgG-free BSA/PBS) for 2 h at room temperature. After washing with 1x PBS for 3 times, sections were mounted with antifade Vectashield mounting media (Vector Laboratories, Burlingame, CA), and signals were visualized under Nikon-E600 fluorescence microscope (Nikon, Tokyo, Japan). Primary antibodies used are: cleaved caspase-3, phospho- and total-ERK, JNK, p38. Fluorescence signals were visualized under Nikon-E600 microscope (Nikon, Tokyo, Japan). DAPI (4′, 6-diamidino-2-phenylindole; Sigma-Aldrich, St. Louis, MO) was used to counterstain nuclei [53].

### TUNEL staining

Terminal deoxynucleotidyl transferase dUTP nick end labeling (TUNEL staining) to detect DNA fragmentation was performed using DeadEnd fluorometric TUNEL kit (Promega, Madison, WI) following manufacturer's protocol [53]. Briefly, paraformaldehyde-fixed paraffin-embedded tumor sections (5 μm thick) were deparaffinized in xylene, rehydrated with sequential immersion in graded ethanol (100%, 95%, 85%, 70%, 50%) and washed with 0.85% NaCl. Tissue sections were fixed with 4% paraformaldehyde/PBS and permeabilized with proteinase K solution (20 μg/ml) for 10 min at room temperature. The nicked DNA was labeled with fluorescence-labeled dUTP nucleotide and terminal deoxynucleotidyl transferase enzyme mix for 60 min at 37 °C. After washing with 2x standard saline citrate solution (SSC) and 1x PBS, slides were mounted with antifade Vectashield mounting media (Vector Laboratories) and sections were examined under fluorescence microscopy (Nikon E-600). Nuclei were counterstained with DAPI (Sigma).

### Hanging-drop assay for spheroid formation

Neuroblastoma cells were prepared as single cell suspension in complete culture medium (DMEM + 10% FBS) without or with metformin (1, 10 and 20 mM). Twenty microliter drop of each prepared cell suspension containing 20,000 cells/drop were pipetted into the inner side of the lid of a 60 mm diameter tissue culture dish [22, 23]. The lid was gently inverted and placed on top of the culture dish, and incubated under tissue culture conditions allowing the cells to form aggregate at the base of the droplet. The image of cells in each droplet was taken by Olympus SZX2 stereo microscope (Olympus, Center Valley, PA).

### Rho GTPase activation assays

Activation of Rho GTPases (RhoA, Rac1 and Cdc42) was measured by affinity precipitation of GTP-bound active form of these proteins [54]. Agarose beads immobilized with Glutathione-S-transferase fused Rhotekin binding domain (GST-RBD) were used to pull-down GTP-RhoA while GST fused PAK binding domain (GST-PBD) were used to pull-down GTP-Rac1 or GTP-Cdc42. Briefly, equal amount of total proteins extracted from tumor tissue were incubated with GST-RBD or GST-PBD agarose beads for 1 h at 4°C, washed with lysis buffer, separated on 14% SDS-polyacrylamide gels and Western blotted for RhoA and Cdc42 (1:500; Santa Cruz Biotechnology) and Rac1 (1:500; BD Biosciences) to detect the GTP-bound form of these proteins. Total RhoA, Rac1 and Cdc42 levels in corresponding cell lysates were also determined by Western blotting for normalization.

### Adenoviral infections

Cells (SH-SY5Y and SK-N-BE(2)) were infected with adenoviruses expressing green fluorescent protein (GFP)-fused constitutively active RhoA (RhoA-V14), dominant negative mutant of Rac1 and Cdc42 (Rac1-N17 and Cdc42-N17) or GFP (control) in serum-free medium at multiplicity of infection (moi) of 100. After 2 h of infection, cells were supplemented with 10% FBS and cultured for an additional 24 h before metformin treatments.

### Data analysis

Data are presented as the mean ± standard deviation of at least three independent experiments. Comparisons were made among the groups using one-way ANOVA followed by Tukey-Kramer ad hoc test (GraphPad software, La Jolla, CA). A *p*-value < 0.05 was considered significant.

## ABBREVIATIONS

b.wt.: body weight
BSA: bovine serum albumin
DAPI: 4′,6-diamidino-2-phenylindole
ERK: extracellular signal-regulated kinase
JNK: c-Jun N-terminal kinase
met: metformin
MAP kinases: mitogen activated protein kinases
TUNEL: terminal deoxynucleotidyl transferase dUTP nick end labeling
