# Correction: Metformin impairs Rho GTPase signaling to induce apoptosis in neuroblastoma cells and inhibits growth of tumors in the xenograft mouse model of neuroblastoma

**DOI:** 10.18632/oncotarget.10404

**Published:** 2016-07-04

**Authors:** Ambrish Kumar, Nadia Al-Sammarraie, Donald J. DiPette, Ugra S. Singh

Present: Due to an error made during the assembly of Figure 6A, the same image panel was inadvertently used for both the RhoA-V14+met (Column 4, Row 1) and Rac1-N17+met (Column 2, Row 2)

Corrected: Correct Figure 6A is provided below. The authors sincerely apologize for this error.

Original article: Oncotarget. 2014; 5(22): 11709-22. doi: 10.18632/oncotarget.2606.

**FIGURE 6:**
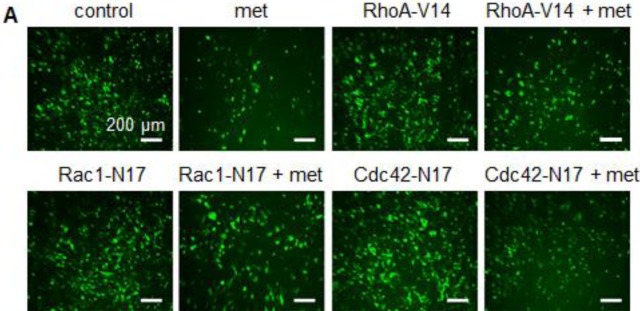
Rho GTPase inhibitors attenuate metformin effects on the survival of neuroblastoma cells (A) Fluorescence images showing the signals of GFP-Rac1, GFP-Cdc42 and GFP-RhoA in SH-SY5Y cells. Cells were infected with adenoviruses expressing GFP-fused dominant–negative Rac1-N17 and Cdc42-N17, and constitutively active RhoA-V14. GFP alone were used as control. After 24 h infection, cells were treated with metformin (10 mM) and further grown for 6 days and photographed. Scale= 200 μm. Number of GFP-containing cells (green) was counted from 10 random fields and plotted.

